# Microbiome yarns: the Global Phenotype–Genotype Survey: Episode II: laryngeal microbiota and vocal phenotypes (*or diction and addiction*)

**DOI:** 10.1111/1751-7915.13375

**Published:** 2019-02-15

**Authors:** Kenneth Timmis, Franziska Jebok, Manfred Rohde, Gabriella Molinari

**Affiliations:** ^1^ Institute of Microbiology Technical University Braunschweig Germany; ^2^ Institute for Educational Science University of Freiburg Freiburg Germany; ^3^ Central Facility for Microscopy Helmholtz Centre for Infection Research Braunschweig Germany

*BBZ, Studio 7A, BBZ Plaza, Burbank, 7.30 pm* 1, 2, 3, 4

*Abigail Repor‐Tastory* 5 *, Discovery Presenter, turns to face the camera*:

Good evening viewers and welcome to a new episode of ‘Discoveries that Change our Lives’. Our guest this evening is once again Dr. Anastasia Noitall‐Most^5^ from the Streber Elite University of Los Angeles.^5^ Good evening Dr. Noitall‐Most *(shaking hands)* and thank you for appearing on the program.

*Dr. Noitall‐Most* Good evening Abi; it is always a pleasure to be here.

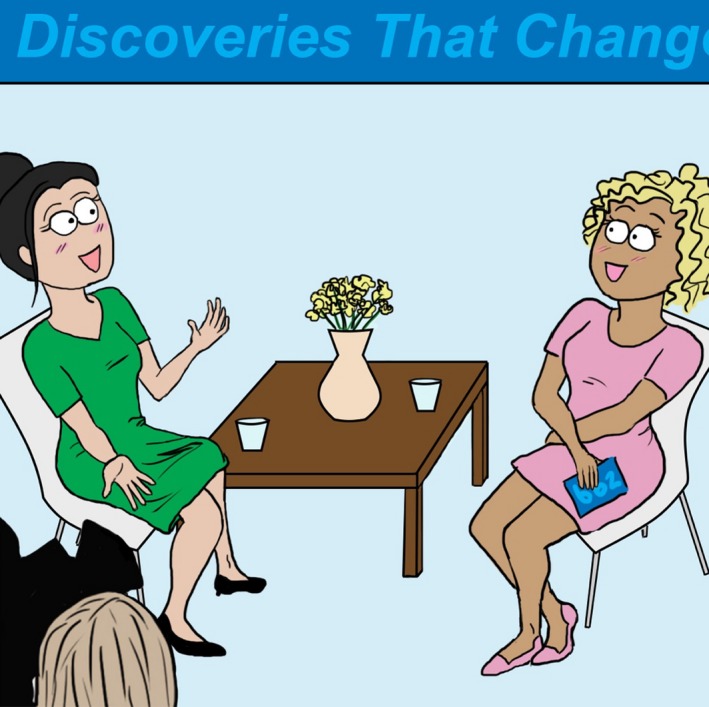

*Ms. Repor‐Tastory* Ani, this evening we will continue to explore the subject of the last episode, namely the most ambitious human microbiome survey to date, the Global Phenotype‐Genotype Survey, or *GLOPS*.^6^

*Dr. Noitall‐Most* Yes: we already knew that our microbiomes seem to influence several health‐related aspects of our biology, but the magnitude and range of human characteristics suggested by the survey to be influenced by the microbiome is just mind boggling. And now, one year later, we have the results of the trials carried out to test the predictions of the correlations found in the survey. The trials indeed established a number of really interesting human characteristics that are significantly influenced by our microbiome. A taste of this was given in the previous programme when I revealed that a new type of bacterium found in the oral cavity seems to influence diction.^6^

*Ms. Repor‐Tastory* Yes, Ani, that was absolutely amazing: please tell us more.

*Dr. Noitall‐Most* Right! This evening I should like to share with viewers new information on the influence of laryngeal microbes on vocal quality.

Firstly, as we know, smokers tend to have gravelly voices and habitually enjoy a recreational, somewhat phlegmy cough‐athon after emerging from their slumber. This comforting, clearing‐of‐the‐throat ritual was previously thought to be due to smoke‐provoked irritation and inflammation of the larynx, and overnight accumulation of laryngeal mucous, but *GLOPS* and subsequent trials based on its findings have consigned this theory to the bin. Metagenome analysis revealed that smokers, but not non‐smokers, have a particular bacterium in their laryngeal microbiota. This finding induced the Imaging Group of Mabriella Golinari and Ranfredy Mohde of the Walpur Gisnacht Institute for Cellular Pathology in Bad Hurzbarg in Northern Germany,^7^ which was responsible for microbe isolation and characterization in *GLOPS*, to attempt to culture and characterise this bug.

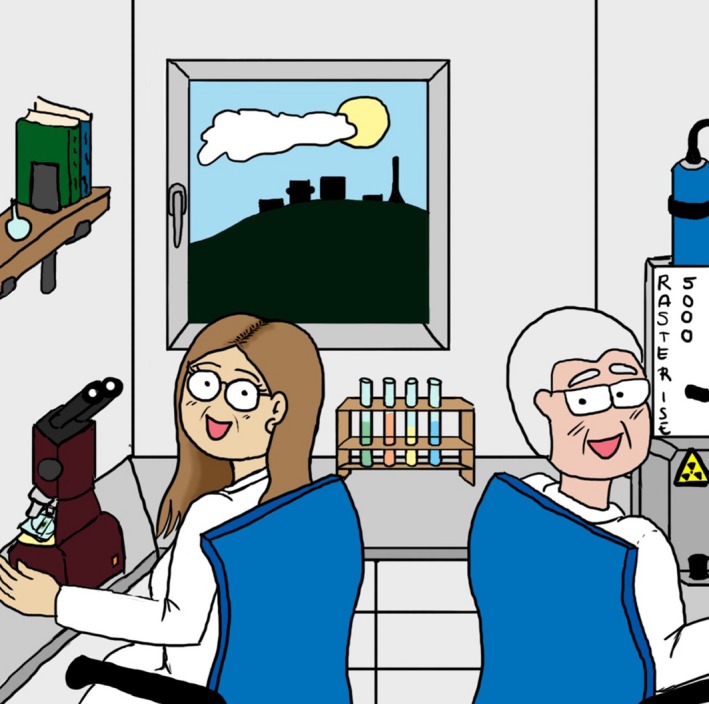

Mab and Ran initially had no success using normal isolation media, but subsequently were able to tease it to grow on a special medium containing nicotine. It subsequently transpired that this microbe is nicotine‐dependent – *yes, really: nicotine‐addicted!* – and able to degrade and use nicotine as a food source.^8^ They named it *Seudomonas nicotinadicta*, which was immediately re‐named *Snica* by young smokers. Mab and Ran showed that *Snica* is distributed throughout the buccal cavity of smokers and is particularly dense on vocal chords.

*Ms. Repor‐Tastory* Gosh: imagine eating nicotine!

*Dr. Noitall‐Most* True – it does not bear thinking about! But of course microbes do not necessarily share our culinary tastes and, in reality, collectively they will eat most anything.

Nevertheless, nicotine is toxic for most bacteria,^9^ as indeed it is to most types of cell, which is why it is so bad for our health.^10^ Apparently, smoking toxicity produces changes in the laryngeal microbial community,^11^ which becomes dominated by *Snica*. *Snica* continuously degrades and removes nicotine from the oral mucosa, and thereby reduces the exposure of other mucosal microbes to nicotine toxicity – a sort of bioprotection service^12^ it provides to the oral microbiota. *Snica* is also resistant to the toxic effects of nicotine, partly due to metabolic and oxidative stress responses,^13^ and partly due to smoke‐induction of the biofilm mode of growth,^13^ which is the *hunker‐down mechanism* microbes adopt when challenged by toxic insults,^13^ such as antibiotics and immune defences, that enables them to avoid and resist antimicrobial activities. But, for the discussion this evening about oral microbiota and vocal quality, a key thing about *Snica* is that it is a prolific slime producer.^14^^,^ 15

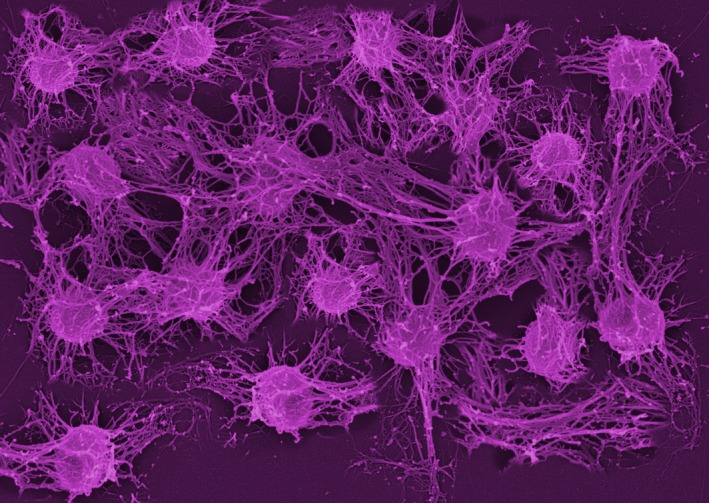

During the day, this slime is unconsciously swallowed, so is not noticeable, but during sleep it accumulates and ultimately triggers the resonant fruity bout of wake‐up‐to‐the‐joys‐of Spring coughing that greets the smoker's daybreak. Importantly for our discussion today, the continual slime production also has the effect of changing the resonance of the vocal chords, such that the sound output has the gravelly timbre typical of the smoker.

*Ms. Repor‐Tastory* Well, that is an eye‐opener: so a slimy bug in the throat is at the bottom of the deep voice!

*Dr. Noitall‐Most* So it would seem. Anyway, staying on the subject of vocal harshness: whereas this is mostly a characteristic of smokers, it is also found in non‐smokers and can be either inherited from a gruffy‐voiced parent, or acquired, for example through perpetual shouting, as evidenced by non‐smoking sergeant majors with typical parade ground timbres. In fact, *GLOPS* did suggest the presence of a non‐*Snica*‐type bacterium in the buccal cavity of non‐smokers with harsh voices that was lacking in those blessed with mellow voices. Encouraged by their success with *Snica*, Mab and Ran wanted to complete their analysis of microbial involvement in vocal harshness and were, with some difficulty, able to isolate this bug. They named it *Grufficus cockeri*, which was immediately re‐christened *Gruffy* by the music industry, and showed it to inhabit principally the surface of the epiglottis and, in some cases, also the vocal chords. It is present in large numbers in people with harsh voices and low numbers in those with euphonius voices. Its very obvious characteristic is that it forms *macrofibres*: when *Gruffy* is growing, newly‐formed cells do not separate but remain attached to one another, forming a long chain which, in time, repeatedly folds back on itself. As cells continue to grow and form daughters, the force generated causes the filament to twist and form a so‐called macrofibre.^16^

Mab and Ran suggest that *Gruffy* macrofibres create a net‐like structure that acts like a dishcloth in the voice box, collecting all manner of flotsam floating and flying around the buccal cavity, and thereby distorting the sounds emanating through the vocal chords, much like the electronic distortion of guitar notes produced by fuzz pedals^17^ and the like.

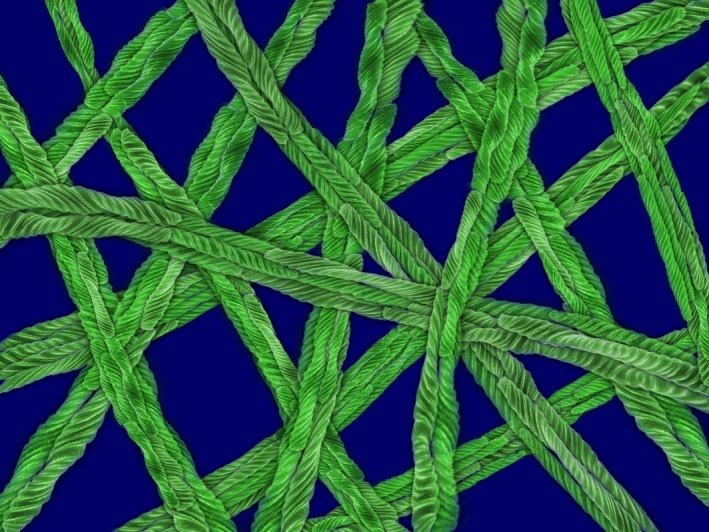

*Ms. Repor‐Tastory* Well, on thinking about it, my throat also feels as if it contains a dishcloth, the morning after a long night of clubbing.

*Dr. Noitall‐Most* Yes, but that is something different and probably your state of dehydration after excessive alcohol consumption.

Anyway, to continue: a limited trial funded by a well‐known music label involved the daily breakfast consumption of yoghurt containing *G. cockeri* by country singers with dulcet voices who were desperate to acquire gravelly voices so that they could sing their preferred music – hard rock or heavy metal – without needing to develop a smoking habit. In almost all cases, within one week, they were singing raucously like Joe Cocker^18^ or Janis Joplin.^19^

*Ms. Repor‐Tastory, amused enlightenment slowly spreading across her face* Oohhh, this explains the sharp deepening of my voice after a wild fling in Nashville with a hunky, husky bluegrass banjo player that took ages…..*……(angry noises emanating from the in‐ear headphone)*…Oh, sorry, I did not mean to interrupt…

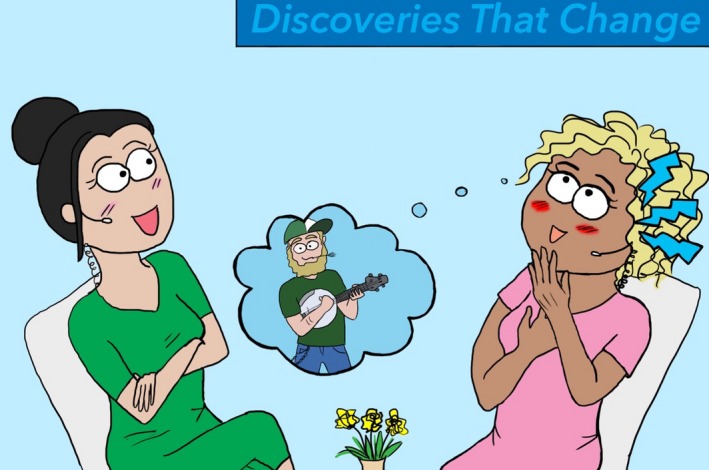

*Dr. Noitall‐Most* Yes, wild flings can be followed by all sorts of surprises, not only deliveries of red roses.

*Ms. Repor‐Tastory, thoughtfully* Hhmmm…

*Dr. Noitall‐Most* But while the transfer of oral bugs between consenting adults is one thing, the unknown and perhaps unwanted transfer of such bugs is another. You will recall that a major focus of *GLOPS* was the transfer of microbiota among people and the phenotypic consequences of this? Well, one aspect of this related to tonight's topic was an assessment of the transfer of airway microbiota by pillows among travellers. You know the situation: those of us who sleep on our fronts or sides breathe, cough, sneeze and dribble into our hotel pillows for 8‐h stretches, vigorously propelling our airway‐oral bounties into their depths. Now while the pillowcases are regularly laundered and changed for every new guest, the pillows generally are not. And, those of us who sleep on our fronts or sides not only blow *our* stuff into pillows, we also inhale and, in the case of pillows used by others, inhale *their* stuff. Previously, it was thought that the post‐travel respiratory issues we not infrequently experience were due to the drying out of the airway mucosa in low humidity aeroplane cabins or to infections gifted by other travellers in confined spaces, like elevators. But now we know that hotel pillows can also be a rich source of diverse microbes that enrich our airway microbiota, sometimes with unexpected, sometimes unwanted, results.

*Ms. Repor‐Tastory* Hhmmm! So: sleeping on our backs, or taking our own favourite pillow, populated with our own microbiota, to enjoy slumber comfort on our travels, also has the benefit of protecting our oral microbiota from invasion by those of others? I think I'll take my own pillow next time I am on the road!

Oh, by the way, do microbes have anything to do with boys’ voices breaking in puberty?

*Dr. Noitall‐Most* Aahhh, that is a fascinating question, but we will leave it for another episode dealing with the Y‐chromosome‐specific microbiota. However, voice breaking is most dramatic when singing, and the survey did make a remarkable discovery about oral microbiota and singing voice purity. Although the samples in the stratification were small, there did seem to be a clear relationship between the presence of a new laryngeal bacterium and voice purity in professional singers, particularly opera singers. This bacterium was readily isolated by Mab and Ran from the buccal cavities of several stars, the identities of which have not been revealed, in anticipation of future commercialisation of this bug and the creation of new brands based on the names of famous singers. Amazingly, when cells of this bacterium were added to vocal chords excised from fresh cadavers, they bound tightly and spontaneously reorganised themselves in such a way that they increased the tension in and caused contraction of the chords. This was slightly disconcerting for Mab and Ran looking down the microscope at this happening in real time, given that the chords were nominally dead! The bug was given the name *Harmonicoccus sublimiae*.^20^

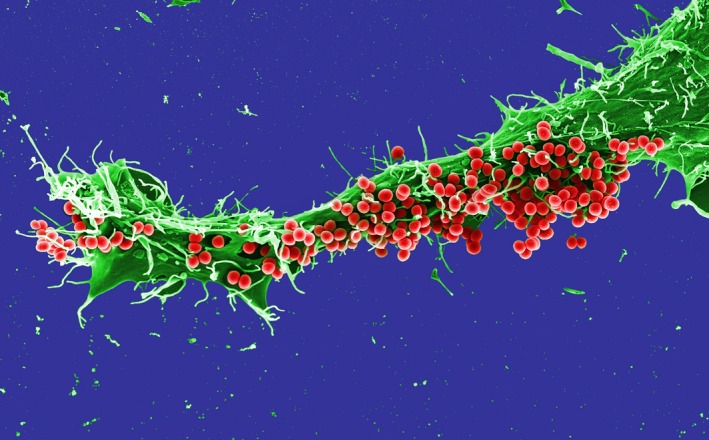

*Ms. Repor‐Tastory* Gosh! Does Harmoniwhatshername‐containing yoghurt improve singing voices? Most Fridays, a close girlfriend and I, both dressed to kill, go to a rather upmarket karaoke bar and it would be nice to have a bit of an edge over the competition….

*Dr. Noitall‐Most* That is of course a key question, but the trial has not yet been concluded.

*Ms. Repor‐Tastory* Hmm – I'll have to get from you the coordinates of the trial leader during our post‐transmission margarita.

*Dr. Noitall‐Most* Certainly. Another rather curious finding was that, rather unexpectedly, individuals involved in the business of persuasion – spin doctors, politicians, lobbyists, ad(wo)men, salespersons – especially those selling automobiles and real estate, industry consultants, fundraisers, gurus of all types, but especially lifestyle and diet gurus, vloggers,^21^ and celebrity chefs, tend to have a somewhat atypical tongue microbiota containing significant numbers of a bacterium that was extremely difficult to isolate in pure culture, though it grew happily in a mixed culture containing other microbes from the buccal cavity. However, the highly experienced German pair finally obtained two isolates by extinction dilution in a rich medium containing traces of silver nitrate. When they characterised the bugs, they found that they have an exceptionally high affinity for silver ions in food and drink consumed,^22^ which they convert to cell‐bound silver nanoparticles,^23^ giving the tongue a slightly silvery appearance. They named them *Silvertonguium guruis,* or *Silg*, and *S. uberdentischzieha*, or *Sudz*.^24^

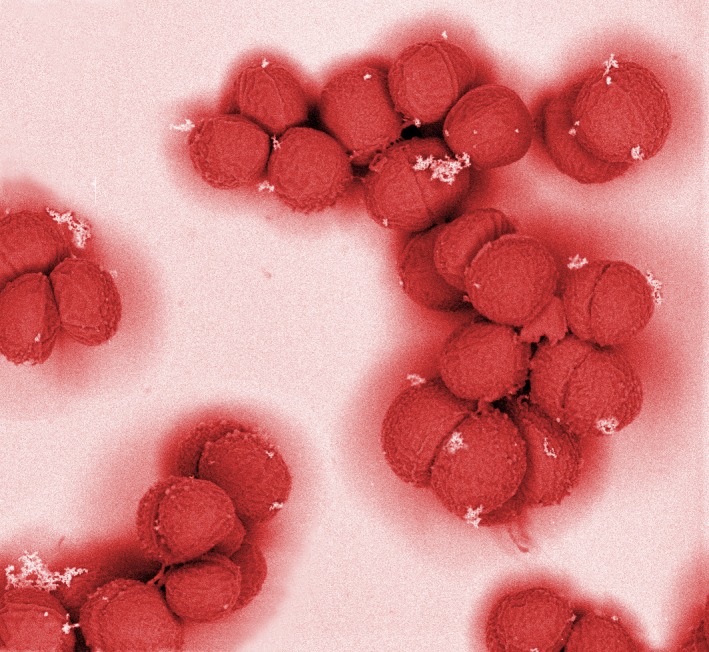

Curious to know if *Silg* and *Sudz* transplantation could improve the rhetorical ability of recipients, a small trial was carried out, but it was quickly aborted when it became apparent that *Silg* and *Sudz* failed to colonize and survive on the tongues of most donors.

*Ms. Repor‐Tastory* So, the adage that people with the power of persuasion have silver tongues is true after all! But why don't transplants of *Silg* and *Sudz* work?

*Dr. Noitall‐Most* Well, the explanation offered by the trial consultant, Professor Tim Kennis of the Queenton Institute of Advanced Studies is that exceptional rhetorical ability in certain individuals^25^ appears to have a genetic component that predetermines not only development of oratorical skills but also predisposes such individuals to lifestyles involving persuading other members of society. He considers it to be entirely plausible that this genetic trait simultaneously modifies normal tongue physiology such that it creates an attractive environment for *Silg* and *Sudz*. To explore this possibility, he carried out a small study on buccal fluids from *Sudz*‐positive and ‐negative individuals and found that *Sudz* grew happily in fluid from *Sudz*‐positive individuals but hardly at all in fluid from *Sudz*‐negative individuals, which supports the view that the tongue physiology of persuaders is different from that normal folk. He has carried out bioinformatic analyses of the genomes of *Sudz* and its relatives and has shown that, in terms of human evolution, silver nanoparticle production by *Sudz* and its predecessors is a relatively ancient trait, but that it is only found in human‐colonising strains, not in animal‐colonising relatives. He suggests that humans have always valued the ability to persuade and direct the views of their fellow men and women, and that the genetic trait that favours this ability has co‐evolved with *Silg/Sudz* ancestors over time to increase the efficacy of this ability. He suspects that if we could sample the tongues of the great orators,^25^ like Aristotle, Cicero, Henry V, etc., we would be able to see this co‐evolution of the human and *Silg/Sudz* ancestral genomes leading to current rhetorical abilities.

*Ms. Repor‐Tastory* Hmm – so our silver tongue‐based rhetorical genealogies have a silver thread!

Ok, so finally, let me come to my traditional closing question: are there any applications of this part of the *GLOPS* investigation?

*Dr. Noitall‐Most* Yes, of course, Abi! Unexpectedly, there is not inconsiderable interest in *Gruffy‐*like macrofibre producers for modulating vocal qualities. There is for example a significant, but previously unrecognised demand from people in the military, law enforcement agencies, team sport training, and the like – both squeaky‐voiced males and mellow‐voiced females – who have all the necessary qualities for rapid advancement in these careers, but do not feel that they have the requisite harsh voice tone and throw needed on the parade ground, rioting streets, football field, etc.

*Ms. Repor‐Tastory, with a wicked look* How strange! I have always had a rather melodic voice and when I was a cheerleader of the SCU^5^ Football team, I never had any problems getting the attention of the team.

*Dr. Noitall‐Most* Yeeeess, well. In addition to this niche market, there are a range of more general applications. First and foremost, nicotine‐degrading bacteria and the enzymes of their catabolic pathways are being used in a variety of ways. These include the use of pathway enzymes for biocatalysis – the use of enzymes to carry out chemical reactions – green chemistry that is environmentally and energetically friendly,^26^ and in the removal of nicotine in tobacco processing wastes,^27^ which can cause environment pollution.

But perhaps most interesting to viewers are the health applications of this work.

As I mentioned earlier, smoking results in significant changes in the buccal flora.^12^ Well, it turns out that it also has an impact on the composition and metabolic activities of the gut microbiota.^28^ Whether or not these changes constitute *dysbioses* of airway and/or gut microbiota and, if so, whether or not they contribute to smoking‐related diseases, is not presently known but is the subject of increasingly active research. For example, smokers have a greater susceptibility to respiratory infections.^29^ While this may well be due to smoke‐caused weakened resilience of the airway epithelium and associated immune defences,^29^ it may instead or additionally be due to the functional consequences of airway microbiota *dysbiosis*.

And, given the physical and functional connectivity of the airway and alimentary microbiota, and the association of a number of health issues with gut microbiota *dysbioses*, it might well be that some of the other health prejudicial consequences of smoking result from air/gut microbiota *dysbioses* it causes. Thus, one type of investigation currently underway by new microbiome enterprises, and especially global food companies, which are increasingly interested in the new prophylaxis and therapy opportunities offered by functional foods, is to determine whether or not transplants of healthy oral microbiota might reduce smoking‐associated diseases and, if so, which ones.

*Ms. Repor‐Tastory* Don't you find it amazing, and somewhat confusing, how blurred the business focus of many large companies has become in recent years? A food company used to satisfy our nutritional requirements, a phone company used to enable us communicate remotely with one another, an informatics company used to help us get on the digital highway, but now their activities are all mixed up – they would probably say strategically integrated – and one way or another are all in the health or entertainment businesses.

*Dr. Noitall‐Most* Absolutely true Abi: the world is indeed changing.

Another topic receiving intense attention is nicotine addiction^30^ which, as viewers may know, is *the leading cause of preventable disease and premature death*.^11^ Its biological basis, involving *inter alia* the so‐called *dopamine reward pathway*, is now well defined but so far attempts to find ways and means of aiding smokers to kick the habit have not yielded the desired outcome.^30^ As a result of *GLOPS*, the Lorenzo von Syntech High Security Institute for Artificial Life in Madrid, headed by the world‐renowned Professor Vic Torde, recently embarked on an ambitious synthetic microbiology programme to design an excellent throat colonising bacterium that will smooth the withdrawal process for those who finally decide that it is high time to quit. This bug, tentatively named by Vic *Nicowithdrawia facilensis*, will contain the following: (i) a highly efficient nicotine degradation pathway to reduce the amount of nicotine entering the lungs during puffing activity, which is intended to have the dual benefit of reducing nicotine‐related tissue damage and of gently weaning smokers off their addiction,^31^ (ii) a new metabolic route for high level production of vitamin C, to compensate for the increased need for vitamin C in smokers,^32^ and (iii) novel biosynthetic pathways for the production of 2 neuroactive compounds that counteract the nicotine withdrawal symptoms of irritability, depressed mood, hedonic dysregulation, restlessness, anxiety, concentration difficulty, increased hunger, insomnia and tobacco craving^32^, ^33^. Experiments with some of the initial constructs given to addicted mice look promising, so smokers may soon have a golden bullet at their disposal.

*Ms. Repor‐Tastory* Well, I never: smoking mice!

*Dr. Noitall‐Most* Vic is also very interested in the nanosilver production activities of *Silg* and *Sudz* because nanosilver has an amazing spectrum of applications in industry, food manufacture, medicine and consumer products^23^, ^34^. An important property of silver nanoparticles is their powerful antimicrobial activity, which is exploited in disinfectants, decontamination, air purification, and the coating of medical devices, like catheters, to prevent their colonisation by microbes, especially pathogens, and for impregnation of clinical clothing. Nanosilver is also used to improve diagnostic imaging and is showing promise as an enhancing agent in cancer therapies. Though he will not be very specific at this stage, Professor Torde is designing derivatives of *Silg* that not only produce silver nanoparticles at much greater efficiencies than natural microbes, but also derivatives that produce functionalised nanoparticles, that is, silver particles coated with molecules, including nanobodies – designed single chain camel antibiodies,^35^ that endow them with high affinities for a diverse range of targets for the purpose of detection, measurement and, in some cases further metabolism or harvesting. I am sure we will learn of some very interesting advances from Vic in the near future.

And while on the topic of *Silg* and *Sudz*, an amazing number of people now routinely have their microbiomes sampled and composition determined, not only to upstage their friends at cocktail parties and on social media, but also to learn about their health‐related genetic profiles. Interestingly, a not insignificant number of people have discovered that they have *Silg* or *Sudz* residing in their buccal mucosa. And not an insignificant number of such individuals pay genome companies small fortunes to carry out genealogy investigations to assess whether they might be descendants of one or other of the great Greek or Roman orators. Most of these do it out of curiosity, but others, especially budding spin doctors, politicians, sales personnel and lobbyists, do it in order to enhance their CVs with tenuous documentation of credentials and pedigrees of their persuasive abilities.

*Ms. Repor‐Tastory* well, viewers: on that rhetorical note, we end this edition of ‘Discoveries that Change our Lives’. We will be back next week with a follow‐up edition to reveal some more of the amazing findings of *GLOPS* and its experimental follow‐up.

*As the camera pulls away, viewers see Abi leaning towards Ani and hear her saying* to get back to my karaoke career aspirations…

++++++++

## Conflict of interest

None declared.

## Footnotes

1 To view previous issues of *Microbiome Discoveries that Change our Lives*, go to http://www.theabsurdmicrobe.com/discoveries-oldstuff.

2 Health Warning. This article is a figment of the authors’ imagination; any serious contemplation may unnecessarily challenge the mental harmony of the reader.

3 Any possible resemblance of the fictional characters and organisations in this fable to a real person, organisation or enterprise is coincidental and unintentional.

4 Dedicated to pioneering researchers of the microbiome, synthetic microbiology, and especially those revealing human phenotypes influenced by our microbial partners, to pioneers of microbial nicotine degradation, especially Rod Brandsch, of biocatalysis, especially to the memory of those heavyweights Andreas Kiener and Bernard Witholt, who have sadly passed on, of nanosilver production and application and, as always, to the memory of Dan Koshland, Jr., father of Dr. Noitall (e.g. see Editorial: A simple proposal for health care rationing. Science 264: 1515), who in turn is the father of Dr. Anastasia Noitall‐Most, and David Jones, father of Daedalus, who invented Dreadco (see, e.g. https://www.newscientist.com/article/mg23531413-500-what-did-daedalus-talk-about-in-septembers-past/; https://www.improbable.com/2017/07/27/sad-news-daedalus-david-jones-is-gone/).

5
http://www.theabsurdmicrobe.com/discoveries/.

6 Timmis, K., Jebok, F., Rohde, M and Molinari, G. (2019) Microbiome yarns: the global phenotype‐genotype survey. Episode I: all my worldly goods, including my microbiome, I thee endow. *Micro Biotechnol* **12**: 11–24.

7 See Timmis, K., Jebok, F., Molinari, G., Rohde, M. and Timmis, J. K. 2017. Microbiome yarns: microbiome basis of memory. *Microbiol Biotechnol* **10:** 1283–1292.

8 Faitelowitz, A. (1927) Bacterial decomposition of tobacco as leading to the formation of bases in the presence of water. *Biochem J* (London) **21**: 262–264; Weber, K. (1953) The decomposition of nicotine during the fermentation of tobacco. *Mitt Gebiete Lebensm u Hyg* **26**: 214–249; Bucherer, H. and Enders, C. 1942. Abbau von Histamin und Nicotin durch Bakterien. *Biochem Z* **310**: 222–224; Wada, E. and Yamasaki, K. (1953) Degradation of nicotine by soil bacteria*. J Am Chem Soc* **76**: 155–157; Brandsch R. (2006) Microbiology and biochemistry of nicotine degradation. *Appl Microbiol Biotechnol*. **69**: 493–8.

9 Keene, K. and Johnson, R. B. (1999) The effect of nicotine on growth of *Streptococcus mutans. Miss Dent Assoc J* **55**: 38–39; Pavia, C. S., Pierre, A. and Nowakowski, J. (2000). Antimicrobial activity of nicotine against a spectrum of bacterial and fungal pathogens. *J Med Micro* **49**: 675–6; Ruan, A.‐D. and Liu, C.‐X. (2015) Analysis of the effect of nicotine on microbial community structure in sediment using PCR‐DGGE fingerprinting. *Water Sci Eng* **8**: 309–314.

10
https://www.humber.nhs.uk/Downloads/Services/Health%20Trainers/Smoking%20Illness%20and%20Death.pdf; Jha, P., Ramasundarahettige, C., Landsman, V. et al (2013) 21st century hazards of smoking and benefits of cessation in the United States. *N Engl J Med* **368**: 341–350; https://www.cdc.gov/tobacco/data_statistics/fact_sheets/health_effects/effects_cig_smoking/index.htm.

11 Charlson, E. S., Chen, J., Custers‐Allen, R. et al. (2010) Disordered microbial communities in the upper respiratory tract of cigarette smokers. Plos One **5**: e15216; Brook, I. (2011) The impact of smoking on oral and nasopharyngeal bacterial flora. *J. Dent. Res* **90**: 704–710; Morris, A., Beck, J. M., Schloss, P. D. et al (2013) Comparison of the respiratory microbiome in healthy non‐smokers and smokers. *Am J Resp Crit Care Med* **187**: 1067–1075; Jette, M. E., Dill‐McFarland, K. A, Hanshew, A. S. et al. (2016) The human laryngeal microbiome : effects of cigarette smoke and reflux. *Sci. Rep*. **6**: article 35882; Wu, J., Peters, B. A., Dominianni, C. et al. (2016) Cigarette smoking and the oral microbiome in a large study of American adults. *ISME J*. **10**: 2435–2446; Yu, G., Phillips, S., Gail, M. H. et al. (2017) The effect of cigarette smoking on the oral and nasal microbiota. *Microbiome* **5**: 3 https://doi.org/10.1186/s40168-016-0226-6

12 e.g. see Erb, R.W., C.A. Eichner, I. Wagner‐Döbler and K.N. Timmis. (1997) Bioprotection of microbial communities from toxic phenol mixtures by a genetically‐designed pseudomonad. *Nature Biotechnology,* **15**: 378–382.

13 Mah, T.‐F. C. and O'Toole, G. A. (2001) Mechanisms of biofilm resistance to antimicrobial agents. *Trends Microbiol* **9**: 34–39; Mihasan M., Chiribau C. B., Friedrich T., Artenie V., Brandsch R. (2007) An NAD(P)H‐nicotine blue oxidoreductase is part of the nicotine regulon and may protect *Arthrobacter nicotinovorans* from oxidative stress during nicotine catabolism. *Appl Environ Microbiol* **73**: 2479–85; Shao, T., Yuan, H., Yan, B. et al. (2009) Antioxidant Enzyme Activity in Bacterial Resistance to Nicotine Toxicity by Reactive Oxygen Species. *Arch Environ Contam Toxicol* **57**: 456–462; Hutcherson, J. A., Scott, D. A. and Bagaitkar, J. (2015) Scratching the surface ‐ tobacco‐induced bacterial biofilms. *Tob Induc Dis*. **13**: 1 https://doi.org/10.1186/s12971-014-0026-3; Tang, H., Zhang, Y., Ma, Y. et al (2018) Regulation of nicotine tolerance by quorum sensing and high efficiency of quorum quenching under nicotine stress in *Pseudomonas aeruginosa* PAO1. *Front Cell Infect Microbiol*. **8**: 88 https://doi.org/10.3389/fcimb.2018.00088.

14 Beckwith, T. D. (1931) The bacteriology of pulp slime. *J Bacteriol* **22**: 15–22; Sanborn, J. R. (1944) Slime‐producing coliform and coliform‐like bacteria. *J Bacteriol* **48**: 211–217.

15 No, sorry, this is not *Seudomonas nicotinadicta*, but rather *Alkanindiges illinoisensis* (Bogan, B. W., Sullivan, W. R., Kayser, K. J. et al (2003) Int J Syst Evol Micro **53**: 1389–1395), which is an altogether pedestrian name for such a gorgeous specimen.

16 Mendelson, N. H. (1999) *Bacillus subtilis* macrofibres, colonies and bioconvection patterns use different strategies to achieve multicellular organization. *Environ Microbiol* 1: 471–477; and no, sorry, this is not *Grufficus cockeri*, but rather *Helicobacter aurati* (Patterson, M. M., Schrenzel, M. D, Feng, Y. et al (2000) J Clin Micro 38: 3722–3728), which is unimaginally beautiful but which has been wilfully manipulated here to resemble macrofibres.

17
https://en.wikipedia.org/wiki/Distortion_(music)

18
https://www.youtube.com/watch?v=6waXtxosJ4A

19
https://www.youtube.com/watch?v=bn5TNqjuHiU

20 No, sorry, this is not *Harmonicoccus sublimiae* contracting a vocal chord but artistic licence exploiting a rather fetching *Staphylococcus aureus* infecting a Hep2 cell.

21 https://en.wikipedia.org/wiki/Vlog#cite_note‐17.

22 https://www.foodsafetymagazine.com/magazine‐archive1/junejuly‐2017/risk‐of‐oligodynamic‐silver‐use‐in‐food‐preservation‐and‐processing‐operations/; https://archive.epa.gov/pesticides/reregistration/web/pdf/4082fact.pdf; https://www.atsdr.cdc.gov/phs/phs.asp?id=537&tid=97.

23 Tran, Q. H., Nguyen, V. Q. and Le, A.‐T. (2013) Silver nanoparticles: synthesis, properties, toxicology, applications and perspectives. *Adv Nat Sci: Nanosci Nanotech* 4: https://doi.org/10.1088/2043-6262/4/3/033001.

24 No, sorry: this is not *Sudz* displaying nanosilver particles, but rather a fine specimen of *Staphylococcus aureus* displaying zirconium oxide nanoparticles.

25 https://www.artofmanliness.com/articles/classical‐rhetoric‐101‐an‐introduction/; https://www.artofmanliness.com/articles/history-of-rhetoric/.

26 Roduit, J.‐P., Wellig, A. and Kiener, A. (1997) Renewable functionalized pyridines derived from microbial metabolites of the alkaloid (S)‐nicotine. *Heterocycles* **45**: 1687–1702; Schenk, S., Hoelz, A., Krauss, B. and Decker. K. (1998) Gene structures and properties of enzymes of the plasmid‐encoded nicotine catabolism of *Arthrobacter nicotinovorans*. *J Mol Biol*. **284**: 1323–39; Wang, S. N., Xu, P., Tang, H. Z. et al. (2005) “Green” Route to 6‐Hydroxy‐3‐succinoyl‐pyridine from (S)‐Nicotine of Tobacco Waste by Whole Cells of a *Pseudomonas* sp. *Environ. Sci. Technol* **39**: 6877–6880; Yu, H., Tang, H. and Xu, P. (2014) Green strategy from waste to value‐added‐chemical production: efficient biosynthesis of 6‐hydroxy‐3‐succinoyl‐pyridine by an engineered biocatalyst. *Sci Rep* **4**: 5397; Wang, W., Xu, P. and Tang, H. (2015) Sustainable production of valuable compound 3‐succinoyl‐pyridine by genetically engineering *Pseudomonas putida* using the tobacco waste. *Sci Rep* **5**:16411; Liu, J., Ma, G., Chen, T. et al. (2015) Nicotine‐degrading microorganisms and their potential applications. *Appl Microbiol Biotechnol*. **99**: 3775–85; de Carvalho, C. C. C. (2017) Whole cell biocatalysts: essential workers from Nature to the industry. *Microb Biotechnol* **10**: 250–263; Yu, W., Wang, R., Li, H. et al (2017) Green route to synthesis of valuable chemical 6‐hydroxynicotine from nicotine in tobacco wastes using genetically engineered *Agrobacterium tumefaciens* S33. *Biotechnol Biofuels* **10**: 288; Fitzpatrick, P. F. (2018) The enzymes of microbial nicotine metabolism. *Beilstein J. Org. Chem*. **14,** 2295–2307.

27 Civilini, M., Domenis, C., Sebastianutto, N. et al (1997) Nicotine decontamination of tobacco agro‐industrial waste and its degradation by micro‐organisms. *Waste Man Res* **15**: 3349–358; Wang, M., Yang, G., Min, H. et al. (2009) Bioaugmentation with the nicotine‐degrading bacterium *Pseudomonas* sp. HF‐1 in a sequencing batch reactor treating tobacco wastewater: Degradation study and analysis of its mechanisms. *Water Res* **43**: 4187–4196.

28 Chakraborty, S., Persaud, V., Vanegas, S. et al. (2014) *Adv Microbiol* **4** : 681–691; Chi, L., Mahbub, R., Gao, B. et al. (2017) Nicotine Alters the Gut Microbiome and Metabolites of Gut‐Brain Interactions in a Sex‐Specific Manner. *Chem Res Toxicol*. **18**: 2110–2119.

29 e.g. see Matsunaga, K., Klein, T. W., Friedman, H. and Yamamoto, Y. (2001) Involvement of nicotinic acetylcholine receptors in suppression of antimicrobial activity and cytokine responses of alveolar macrophages to Legionella pneumophila infection by nicotine. *J Immunol*. **167**: 6518–24; Bagaitkar, J., Demuth, D. R. and Scott, D. A. (2008) Tobacco use increases susceptibility to bacterial infection. *Tob Induc Dis* **4**: 12. https://doi.org/10.1186/1617-9625-4-12; Garmendia, J., Morey, P. and Bengoechea, J. A. (2012) Impact of cigarette smoke exposure on host‐bacterial pathogen interactions. *Eur Respir J* **39**: 467–477; and, for an excellent depiction of lung immune defences, see www.nature.com/ni/multimedia/lung and https://www.nature.com/articles/ni.3069.

30 Benowitz, N. L. (2010) Nicotine addiction. *N Engl J Med* **362**: 2295–2303; Jiloha, R. C. (2010) Biological basis of tobacco addiction: implications for smoking cessation treatment. *Ind J Psych* **52**: 301–307

31 see also Xue, S., Schlosburg, J. E. and Janda, K. D. (2015) A new strategy for smoking cessation: characterization of a bacterial enzyme for the degradation of nicotine. *J Am Chem Soc* **137**: 10136–9; Xue, S., Kallupi, M., Zhou, B. et al (2018) An enzymatic advance in nicotine cessation therapy. *Chem Commun (Camb)*. **54**: 1686–1689.

32 Schectman, G., Byrd, J. C. and Gruchow, H. W. (1989) The influence of smoking on vitamin C status in adults. *Am J Public Health*. **79**: 158–62; Lo, J., Schabel, M. C., Roberts, V. H. et al (2015) Vitamin C supplementation ameliorates the adverse effects of nicotine on placental hemodynamics and histology in nonhuman primates. *Am J Obstet Gynecol*. **212**: 370.e1–8. https://doi.org/10.1016/j.ajog.2014.12.042.

33 Gourlay, S. G. and Benowitz, N. L. (1995) Is clonidine an effective smoking cessation therapy? *Drugs* **50**:197–207; Coe, J.W., Brroks, P. R., Vetelino, M. G. et al (2005) Varenicline: an alpha4beta2 nicotine receptor partial agonist for smoking cessation. *J Med Chem* **48:** 3473–7.

34 Ge, L., Li, Q, Wang, M. et al (2014) Nanosilver particles in medical applications: synthesis, performance, and toxicity. *Int J Nanomedicine*. 9: 2399–407; Zhang, X. F., Liu, Z.G., Shen, W. and Gurunathan, S. (2016) Silver Nanoparticles: Synthesis, Characterization, Properties, Applications, and Therapeutic Approaches. *Int J Mol Sci*. 17:1534. https://doi.org/10.3390/ijms17091534; Marassi, V., Di Cristo, L., Smith, S. G. J. et al. (2018) Silver nanoparticles as a medical device in healthcare settings: a five‐step approach for candidate screening of coating agents. *R Soc Open Sci*. 5: 171113. https://doi.org/10.1098/rsos.171113; Turner, R J (2018) Is silver the ultimate antimicrobial bullet? *Antibiotics* 7: 112; see also Special Issue: Silver‐Based Antimicrobials (2018) *Antibiotics* 7, issue 4.

35 Chakravarty, R., Goel, S., and Cai, W. (2014) Nanobody: the “Magic Bullet” for molecular imaging? *Theranostics* 4: 386–398.Pinero‐Lambea, C., Bodelon, G., Fernandez‐Perianez, R. et al (2015) Programming controlled adhesion of *E. coli* to target surfaces, cells, and tumors with synthetic adhesins. *ACS Synth Biol* 4: 463–473; Van Audenhove, I., and Gettemans, J. (2016) Nanobodies as versatile tools to understand, diagnose, visualize and treat cancer. EBioMedicine 8: 40–48; Steeland, S., Vandenbroucke, R.E., and Libert, C. (2016) Nanobodies as therapeutics: big opportunities for small antibodies. *Drug Discov Today* 21: 1076–1113; Timmis, K., Timmis, J. K., Brüssow, H. and Fernandez, L. A. (2019) Synthetic consortia of nanobody‐coupled and formatted bacteria for prophylaxis and therapy interventions targeting microbiome dysbiosis‐associated diseases and co‐morbidities. *Micro Biotech* 12: 58–65

